# Trends in admissions and costs for neonatal intensive care in US children’s hospitals, 2017–2022

**DOI:** 10.1038/s41372-026-02619-8

**Published:** 2026-03-17

**Authors:** Ashlee J. Vance, Troy Richardson, Brian King, Joanne Lagatta, Kuan-Chi Lai, Ashwini Lakshmanan, Henry C. Lee, Tamorah Lewis, Ravi M. Patel, Joseph G. Kohne

**Affiliations:** 1 Center for Health Policy and Health Services Research, Henry Ford Health, Detroit, MI USA; 2 Department of Pediatrics, College of Human Medicine, Michigan State University, East Lansing, MI USA; 3 Children’s Hospital Association, Lenexa, KS USA; 4 Department of Pediatrics, Beth Israel Deaconess Medical Center, Harvard Medical School, Boston, MA USA; 5 Department of Pediatrics, Medical College of Wisconsin, Milwaukee, WI USA; 6 Division of Neonatology, Children’s Hospital Los Angeles, Los Angeles, CA USA; 7 Department of Health Systems Science, Bernard J. Tyson Kaiser Permanente School of Medicine, Pasadena, CA USA; 8 Department of Pediatrics, University of California San Diego, La Jolla, CA USA; 9 Department of Paediatrics, University of Toronto Temerty Faculty of Medicine, Toronto, ON USA; 10 Division of Clinical Pharmacology & Toxicology, The Hospital for Sick Children, Toronto, ON USA; 11 Department of Pediatrics, Emory University, Children’s Healthcare of Atlanta, Atlanta, GA USA; 12 School of Medicine, Case Western Reserve Univeristy, Cleveland, OH USA; 13 Pediatric Critical Care, Rainbow Babies and Children’s Hospital, Cleveland, OH USA

**Keywords:** Epidemiology, Health services

## Abstract

**Objectives:**

To evaluate trends in neonatal intensive care unit (NICU) admissions, illness acuity, and hospitalization costs among U.S. children’s hospitals from 2017 to 2022.

**Methods:**

This retrospective cohort study used data from the Pediatric Health Information System (PHIS) and included all NICU admissions. We examined trends in patient characteristics (e.g., gestational age, complex chronic conditions), illness severity scores, and standardized hospital costs. Interrupted time series analyses assessed changes in NICU costs over time.

**Results:**

There were 234,571 infants admitted to NICUs in US children’s hospitals between 2017 and 2022. NICU admissions shifted significantly by gestational age with admissions increasing by 10–18% among extremely, very, and late preterm infants. Term infants remained the largest admission group, averaging 45% annually (*p* < 0.001). Infants with ≥1 complex chronic condition increased from 46.6% in 2017 to 50.9% in 2022 (*p* < 0.001). NICU-specific illness severity increased modestly over time (1.14–1.24, *p *< 0.001). Median standardized NICU hospital costs rose by approximately 20% over the study period, with notable inflections in early 2020 and 2021.

**Conclusions:**

Among US children’s hospitals in the PHIS database, NICU admissions were increasingly complex and costly. These findings highlight the importance of ongoing monitoring of resource use and patient mix in specialized NICU settings.

## Introduction

Neonatal intensive care units (NICUs) play a critical role in managing the care of high-risk infants, with children’s hospitals frequently serving as referral centers for the most complex neonatal conditions. Approximately 10% of all live births in the United States (US) require admission to a NICU with conditions ranging from brief, self-limited illnesses to those requiring prolonged, resource-intensive support [1, 2]. Over the past decade, NICU admission rates have increased by 37% [1]. However, this expansion in care comes with significant costs and longer hospital stays [3–6], with more than 30% of NICU graduates experiencing chronic health needs post-discharge [7, 8]. As survival improves and the population of NICU graduates becomes more medically complex, it is essential to examine how care needs are evolving in NICU care.

In the United States, there are more than 1400 NICUs with approximately 35,000 beds, most of which are classified as Level III or IV. Higher-acuity NICUs with greater bed capacity are concentrated in children’s and academic hospitals and often located in regions with higher population density [9]. Monitoring epidemiologic trends in NICU admissions is important to understand neonatal care’s evolving landscape and identifying emerging challenges and patterns, given their critical role in the care of high-risk newborns and infants [10]. Previous research has primarily focused on preterm birth and the associated direct medical costs [11, 12], often overlooking the significant proportion of NICU admissions that involve late preterm and full-term infants [13]. For example, between 2016 and 2023, NICU admission rates increased by 9% for full-term and 4% for late-preterm infants [14]. Furthermore, few studies have captured trends in illness severity, complex chronic conditions (CCCs), and length of stay (LOS) within US children’s hospitals, where the most intensive neonatal care is delivered. Such data would be particularly useful given the regionalization of NICU care and increasing complexity.

While NICU utilization and the associated costs have risen nationally, granular trend analyses are limited, particularly for high-acuity tertiary settings. Tertiary children’s hospitals offer a unique vantage point into shifts in neonatal case mix and intensity due to their specialization in complex care, although their neonatal populations may differ from other community hospitals. By focusing on these hospitals [15], our study aims to characterize trends in NICU admissions, patient acuity, and hospitalization costs over a five-year period, including the onset and early recovery phases of the COVID-19 pandemic. These findings may inform planning and resource allocation within tertiary care systems and generate hypotheses for broader population-based research. Thus, we sought to explore trends in NICU admission rates and hospital costs among a cohort of infants at US children’s hospitals between 2017 and 2022.

## Methods

### Design and data source

We conducted a retrospective cohort study using data from the Pediatric Health Information System (PHIS), an administrative database maintained by the Children’s Hospital Association (Lenexa, KS). PHIS includes inpatient, emergency department, ambulatory surgery, and observation unit data from 48 tertiary children’s hospitals in the United States. The data available in the PHIS dataset includes patient demographic characteristics, procedure codes, and detailed billing information.

### Guidelines and ethics statement

This study followed the Strengthening the Reporting of Observational Studies in Epidemiology (STROBE) [16] reporting guidelines for cross-sectional studies and determined to be non-human subjects’ research by the Henry Ford Health Institutional Board and exempt from review.

### Study population

**Fig. 1:**
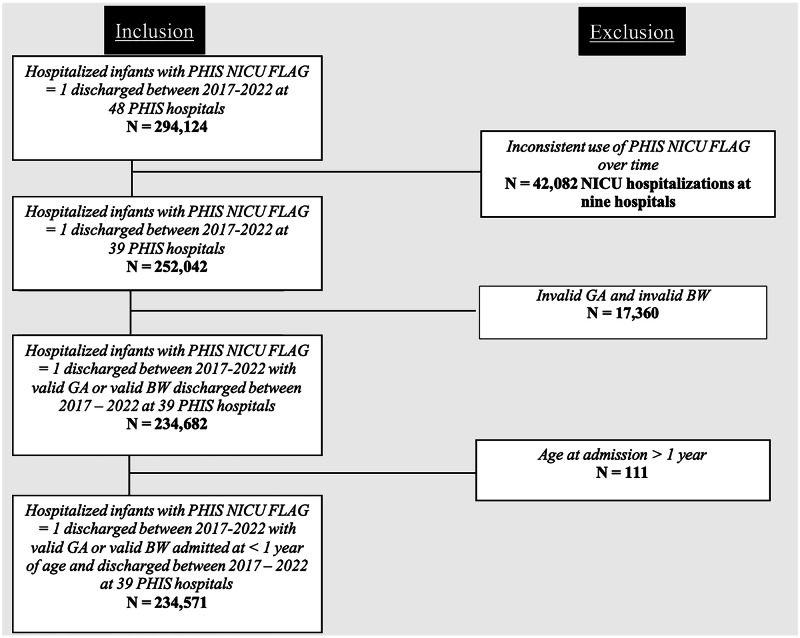
Flow diagram of NICU cohort.

The study cohort included all infants admitted to a PHIS hospital with at least a single day’s room charge for a NICU bed. Infants were included if their discharge date was between January 1, 2017, and December 31, 2022. Encounters were excluded if they originated from a hospital that did not consistently report NICU charges (*n* = 9 hospitals), or if infants had missing or unknown gestational age or birth weight values. Additionally, children with a recorded NICU charge whose age at admission was greater than 1 year were excluded (see Fig. 1). This exclusion criteria were necessary to confirm the cohort included only those patients who were admitted to the NICU immediately following birth. Infant medical complexity was identified and flagged by diagnosis codes using the *Complex Chronic Conditions Classification (CCC) System* [17]. This system identifies conditions using codes from the International Classification of Diseases, 10th Revision (ICD-10), and categorized into 10 domains, and widely adopted for research in pediatric populations for over two decades and shown to correlate with healthcare use and mortality risk [18, 19]. The domains of the CCC are allocated by body system. For instance, the ‘cardiovascular domain’ includes all congenital heart defects and conditions related to the heart. The ‘congenital or genetic defect domain’ includes those conditions not otherwise specified in another domain. Examples of CCC diagnoses for each domain are available in supplementary content.

### Study outcomes

Patient characteristics included gestational age, birth weight, sex assigned at birth, race/ethnicity, insurance type, discharge disposition, admission source, NICU length of stay (LOS) and Child Opportunity Index (COI). Admission source was defined as follows: ‘inborn’ includes infants admitted to the same facility where they were born versus ‘outborn’ includes infants born at a different facility and transferred to a PHIS hospital with a NICU. The COI is an area-level variable that assesses neighborhood conditions, resources, and environments contributing to healthy development [20]. Illness severity was measured using an adapted, H-RISK score, a PHIS-specific metric that assigns a severity score based on the deviation in length of stay for each All Patient Refined Diagnosis Related Group (APR-DRG) compared to expected values. Increasing values of the score indicate increasing mean NICU LOS for the assigned APR-DRG, suggestive of higher severity of illness [21]. This is a standardized method for stratifying illness severity within PHIS data and has been used in prior neonatal research [21]. Hospital characteristics included admissions rates, hospital length of stay, and NICU-related hospital and total hospitalization costs.

### Hospitalization costs

NICU costs were defined as direct medical costs attributable to the NICU stay, from the hospital perspective [22]. Hospital-specific and department-specific cost-to-charge ratios (CCR) were applied to estimate costs, excluding physician fees and indirect costs. CCR are the estimated cost incurred by the hospital. The PHIS dataset uses a standardized unit cost, which is not a direct measure of hospital cost dollars, but a measure of volume related to resources consumed [23]. This standardized unit cost is calculated based on unique hospital median costs for services and developed as a proxy for true resource use that is comparable across hospitals [24]. To adjust for regional variation, costs were standardized using the Centers for Medicare & Medicaid Services wage/price index and inflated to 2022 US dollars using the Producer Price Index for Inpatient Services [25].

### Statistical analysis

Patient and hospitalization characteristics were described using frequencies and percentages for categorical variables, medians with interquartile range (IQR) for non-normal continuous variables and means with standard errors (SE) for continuous variables following a normal distribution. Categorical patient and hospitalization characteristics over time were analyzed using generalized linear models (GLM), which included year as the only variable. We assumed a binomial or multinomial distribution with a respective logit or generalized logit link function for categorical variables and a normal or log normal distribution for continuous variables. We also performed an unadjusted time trend analysis of illness severity assuming a Gamma distribution with log link and NICU-related costs with a log-linear model. We summarized NICU admissions, costs, and prevalence of complex diagnoses by gestational age. Gestational age stratification included extremely preterm (22–27 weeks), very preterm (28–33 weeks), late preterm (34–36 weeks), and term ( ≥ 37 weeks). Finally, to assess trends in healthcare costs, we performed an interrupted time series analysis. We a-priori identified discharges for the calendar year 2020 as the interruption period, representing the unique period of COVID-19 and its impacts on the health system. Because NICU-related costs in 2020 were clearly higher than other years, we compared the trend in geometric mean NICU-related costs pre-2020 with the trend in geometric mean NICU-related costs post-2020 using a log-linear interrupted time series (ITS) approach, ignoring NICU-related costs in 2020. Our ITS model included an effect for a pre-2020 slope estimate, an effect for a stepwise change in model intercept at the point of interruption, and an effect for a post-2020 slope estimate. We performed ITS modeling for the entire cohort as well as by gestational age stratification. All statistical analyses were performed using SAS v9.4 (Cary, NC). *P* values less than 0.05 were considered statistically significant.

## Results

### Cohort trends

A total of 234,571 infants were admitted to NICUs across 39 US children’s hospitals between 2017 and 2022. Demographic and clinical characteristics are summarized in Table 1. Across the 6-year study period, there were significant changes in the gestational age at birth distribution (*p* < 0.001). The proportion of infants with unknown gestational age declined from 18.1 to 10.9%. When evaluating absolute numbers for NICU admission across known gestational ages, admissions increased by 10.3% for extremely preterm infants (from 2753 to 3037), 16.0% for very preterm infants (from 5711 to 6626), and 17.8% for late preterm infants (from 7265 to 8556). Term infants remained the largest single category of admissions by volume, averaging 45% annually. The sex distribution remained stable, with approximately 55.7% of infants identified as male. There were statistically significant changes in documented race and ethnicity (*p* < 0.001), with slight increases in Hispanic infants (16.3% in 2017 to 18.8% in 2022) and decreases in the proportion of infants with unknown race/ethnicity (9.0–6.1%). The complexity of the NICU population also increased. Infants without complex chronic conditions (CCC) declined from 53.4% in 2017 to 49.1% in 2022, while those with ≥2 CCCs increased from 16.9% to 18.2% and infants with ≥4 CCCs rose from 4.7% in 2017 to 5.7% in 2022. Overall, infants with ≥1 CCCs increased from 46.6% in 2017 to 50.9% in 2022 (*p* < 0.001). Table 2 summarizes the prevalence of medically complex diagnoses by gestational age and CCC domain. The most frequent CCC diagnoses were in the neonatal, cardiovascular, and gastrointestinal domains. Cardiovascular diagnoses were the most prevalent CCC category among very preterm (14.5%), late preterm (11.8%), and term infants (14.2%). Among infants born very preterm, gastrointestinal and respiratory CCC diagnoses increased significantly (*p* < 0.001), data not shown. Among late preterm and term infants, the prevalence of renal and urologic conditions rose over the study period (*p* < 0.001), data not shown. Illness severity, measured by the adapted H-RISK score, rose from a mean of 1.14 (SE: 0.01) to 1.24 (SE: 0.01) (*p* < 0.001).

**Table 1:** Infant demographics of NICU admissions in US children’s hospitals from 2017 to 2022.

		Admission Year						*P* value
*N* (%) or median (IQR)	Total	2017	2018	2019	2020	2021	2022	
**NICU Admissions**	234,571	39,614	37,191	39,496	37,487	40,681	40,102	
**Gestational Age (weeks)**								*p* < 0.001
Extremely Preterm ( < 27)	17,042 (7.3)	2753 (6.9)	2609 (7.0)	2873 (7.3)	2724 (7.3)	3046 (7.5)	3037 (7.6)	
Very Preterm (28–33)	36,608 (15.6)	5711 (14.4)	5783 (15.5)	6131 (15.5)	5784 (15.4)	6573 (16.2)	6626 (16.5)	
Late preterm (34–36)	47,627 (20.3)	7265 (18.3)	7469 (20.1)	8078 (20.5)	7615 (20.3)	8644 (21.2)	8556 (21.3)	
Term ( > 37)	104,938 (44.7)	16,720 (42.2)	17,730 (47.7)	18,426 (46.7)	16,825 (44.9)	17,722 (43.6)	17,515 (43.7)	
Unknown	28,356 (12.1)	7165 (18.1)	3600 (9.7)	3988 (10.1)	4539 (12.1)	4696 (11.5)	4368 (10.9)	
**Sex**								*p* = 0.206
Female	103,768 (44.2)	17,441 (44.0)	16,458 (44.3)	17,442 (44.2)	16,516 (44.1)	18,060 (44.4)	17,851 (44.5)	
Male	130,578 (55.7)	22,124 (55.8)	20,677 (55.6)	22,030 (55.8)	20,944 (55.9)	22,587 (55.5)	22,216 (55.4)	
**Race/Ethnicity**								*p* < 0.001
Non-Hispanic White	103,183 (44.0)	17,864 (45.1)	16,402 (44.1)	17,266 (43.7)	16,118 (43.0)	17,968 (44.2)	17,565 (43.8)	
Non-Hispanic Black	45,565 (19.4)	7275 (18.4)	7229 (19.4)	7642 (19.3)	7707 (20.6)	7985 (19.6)	7727 (19.3)	
Hispanic	40,150 (17.1)	6458 (16.3)	5993 (16.1)	6318 (16.0)	6440 (17.2)	7401 (18.2)	7540 (18.8)	
Asian/ Hawaiian /Am. Indian	8528 (3.6)	1443 (3.6)	1343 (3.6)	1396 (3.5)	1338 (3.6)	1515 (3.7)	1493 (3.7)	
Multiple/Other	18,938 (8.1)	3007 (7.6)	2842 (7.6)	3201 (8.1)	3218 (8.6)	3332 (8.2)	3338 (8.3)	
Unknown	18,207 (7.8)	3567 (9.0)	3382 (9.1)	3673 (9.3)	2666 (7.1)	2480 (6.1)	2439 (6.1)	
**Admission Source**								*p* < 0.115
Inborn	52,436 (22.4)	9708 (24.5)	7884 (21.2)	8118 (20.6)	8260 (22.0)	9737 (23.9)	8729 (21.8)	
Outborn	161,060 (68.7)	26,179 (66.1)	26,403 (71.0)	28,095 (71.1)	25,635 (68.4)	27,155 (66.8)	27,593 (68.8)	
Other	21,075 (9.0)	3727 (9.4)	2904 (7.8)	3283 (8.3)	3592 (9.6)	3789 (9.3)	3780 (9.4)	
**Insurance type**								*p* < 0.001
Commercial	93,015 (39.7)	15,482 (39.1)	14,553 (39.1)	15,785 (40.0)	14,296 (38.1)	16,303 (40.1)	16,596 (41.4)	
Government	131,715 (56.2)	22,817 (57.6)	21,252 (57.1)	22,046 (55.8)	21,628 (57.7)	22,453 (55.2)	21,519 (53.7)	
Self-Pay	4,907 (2.1)	729 (1.8)	751 (2.0)	916 (2.3)	744 (2.0)	850 (2.1)	917 (2.3)	
Other	4,934 (2.1)	586 (1.5)	635 (1.7)	749 (1.9)	819 (2.2)	1075 (2.6)	1070 (2.7)	
**Child Opportunity Index**								*p* = 0.239
Very Low	56,517 (24.1)	9609 (24.3)	9219 (24.8)	9743 (24.7)	9311 (24.8)	9431 (23.2)	9204 (23.0)	
Low	49,907 (21.3)	8342 (21.1)	8153 (21.9)	8487 (21.5)	8070 (21.5)	8524 (21.0)	8331 (20.8)	
Moderate	50,322 (21.5)	8866 (22.4)	7799 (21.0)	8283 (21.0)	8045 (21.5)	8799 (21.6)	8530 (21.3)	
High	37,965 (16.2)	6178 (15.6)	5869 (15.8)	6261 (15.9)	5929 (15.8)	6853 (16.8)	6875 (17.1)	
Very High	38,883 (16.6)	6436 (16.2)	6002 (16.1)	6521 (16.5)	5987 (16.0)	6924 (17.0)	7013 (17.5)	
**Complex Chronic Conditions**								*p* < 0.001
0	118,818 (50.7)	21,140 (53.4)	19,221 (51.7)	20,249 (51.3)	18,496 (49.3)	20,031 (49.2)	19,681 (49.1)	
1	62,585 (26.7)	10,027 (25.3)	9761 (26.2)	10,486 (26.5)	10,262 (27.4)	11,227 (27.6)	10,822 (27.0)	
2	27,267 (11.6)	4391 (11.1)	4240 (11.4)	4554 (11.5)	4454 (11.9)	4824 (11.9)	4804 (12.0)	
3	13,679 (5.8)	2208 (5.6)	2120 (5.7)	2214 (5.6)	2211 (5.9)	2427 (6.0)	2499 (6.2)	
4+	12,222 (5.2)	1848 (4.7)	1849 (5.0)	1993 (5.0)	2064 (5.5)	2172 (5.3)	2296 (5.7)	
**Illness severity** (adapted H-risk)	1.19 (0.00)	1.14 (0.01)	1.18 (0.01)	1.17 (0.01)	1.21 (0.01)	1.19 (0.01)	1.24 (0.01)	*p* < 0.001
**LOS** (days)								*p* < 0.001
<7 days	100,360 (42.8)	18,139 (45.8)	16,105 (43.3)	17,139 (43.4)	15,737 (42.0)	16,946 (41.7)	16,294 (40.6)	
8–30 days	80,241 (34.2)	12,902 (32.6)	12,650 (34.0)	13,513 (34.2)	12,897 (34.4)	14,231 (35.0)	14,048 (35.0)	
>31 days	53,970 (23.0)	8573 (21.6)	8436 (22.7)	8844 (22.4)	8853 (23.6)	9504 (23.4)	9760 (24.3)	
**NICU LOS** (days)								*p* < 0.001
< 7 days	119,810 (51.1)	21,276 (53.7)	19,229 (51.7)	20,281 (51.3)	18,625 (49.7)	20,621 (50.7)	19,778 (49.3)	
8 – 30 days	71,212 (30.4)	11,511 (29.1)	11,211 (30.1)	12,054 (30.5)	11,460 (30.6)	12,488 (30.7)	12,488 (31.1)	
> 31 days	43,549 (18.6)	6827 (17.2)	6751 (18.2)	7161 (18.1)	7402 (19.7)	7572 (18.6)	7836 (19.5)	
**NICU costs** (adjusted to 2022 USD)								
Cost per NICU Hospitalization, median (IQR)	$28,052 ($9,484, $83,028)	$24,383 ($7,670, $73,574)	$27,013 ($9,340, $76,985)	$28,216 ($9,900, $83,613)	$31,507 ($10,332, $100,624)	$28,008 ($9,449, $81,938)	$29,234 ($9,953, $85,152)	*p* < 0.001
Cost per NICU Day, median (IQR)	$3470 (3115, 4192)	$3332 (3008, 4056)	$3397 (3067, 4158)	$3564 (3212, 4254)	$3661 (3290, 4407)	$3443 ($3091, 4156)	$3388 ($3041, 4081)	*p* < 0.001
Hospital costs attributable to NICU (%)	23.6	18.9	18.2	20.5	28.1	19.0	19.8	*p* < 0.001
**Mean (SE) NICU costs** (adjusted to 2022 USD)								
Cost per NICU Hospitalization, Mean (SE)	$83,236 ($355)	$73,633 ($785)	$75,681 ($798)	$78,682 ($814)	$90,489 ($929)	$85,132 ($861)	$95,512 ($991)	*p* < 0.001
Cost per NICU Day, Mean (SE)	$3911 ($6)	$3599 ($12)	$3764 ($12)	$3816 ($11)	$4081 ($21)	$4006 ($15)	$4194 ($12)	*p* < 0.001
**Geometric Mean (95% CI) NICU costs** (adjusted to 2022 USD)								
Cost per NICU Hospitalization, Geometric Mean (95% CI)	$27,268 ($27,102, $27,434)	$22,307 ($21,980, $22,639)	$25,976 ($25,584, $26,375)	$26,760 ($26,367, $27,158)	$30,184 ($29,730, $30,646)	$28,073 ($27,667, $28,485)	$31,283 ($30,827, $31,745)	*p* < 0.001
Cost per NICU Hospitalization, Geometric Mean (95% CI)	$3476 ($3,469, $3483)	$3068 ($3054, $3082)	$3381 ($3364, $3397)	$3430 ($3414, $3446)	$3669 ($3652, $3687)	$3574 ($3558, $3590)	$3779 ($3762, $3797)	*p* < 0.001

**Table 2:** Prevalence of medically complex diagnosis by gestational age, n (%).

	Total Cohort (*n* = 234,571)	Extremely Preterm^a^ (*n* = 17,042)	Very Preterm (*n* = 36,608)	Late Preterm (*n* = 47,627)	Term (*n* = 104,938)
**Cardiovascular**	34,461 (14.7)	–	5321 (14.5)	5599 (11.8)	14,951 (14.2)
**Congenital or Genetic Defect**	17,970 (7.7)	–	2173 (5.9)	4230 (8.9)	9022 (8.6)
**Gastrointestinal**	26,679 (11.4)	–	4222 (11.5)	4566 (9.6)	9844 (9.4)
**Hematologic or Immunologic**	4294 (1.8)	–	1024 (2.8)	684 (1.4)	1348 (1.3)
**Malignancy**	1467 (0.6)	–	321 (0.9)	267 (0.6)	570 (0.5)
**Metabolic**	6414 (2.7)	–	1267 (3.5)	995 (2.1)	2034 (1.9)
**Neurological**	19,691 (8.4)	–	3057 (8.4)	3126 (6.6)	8025 (7.6)
**Premature & Neonatal**	70,665 (30.1)	17,042 (100.0)	11,651 (31.8)	9427 (19.8)	26,167 (24.9)
**Renal & Urologic**	16,846 (7.2)	–	2643 (7.2)	3346 (7.0)	7209 (6.9)
**Respiratory**	15,557 (6.6)	–	2662 (7.3)	2431 (5.1)	5969 (5.7)

^a^Infants born 22–26 weeks’ gestation are only included in the premature & neonatal domain.

### Outcome trends

For admissions ≥31 days, hospital length of stay (LOS) (range 21.6–24.3%) and NICU-specific LOS (range 17.2–19.5%) increased across the study period (*p* < 0.001) (Table 1). The proportion of infants admitted for fewer than 7 days in the NICU declined from 45.8 to 40.6% over time. Discharge disposition remained stable over the study period, with approximately 89.2% of infants discharged home each year and in-hospital mortality ranged from 3.5 to 3.7% across all years, data not shown.

### NICU cost trends

**Fig. 2:**
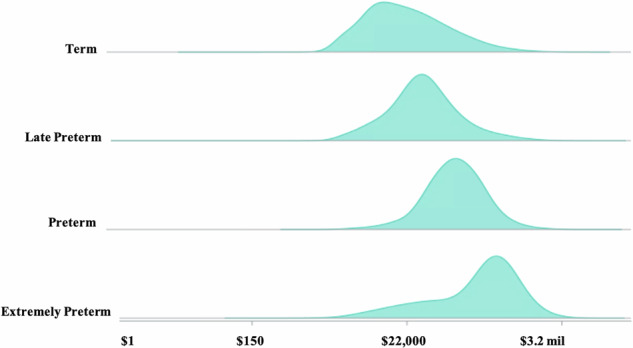
Probability distribution of total NICU hospitalization costs by gestational age.

Total NICU-related costs increased significantly between 2017 and 2022 (*p* < 0.001) (Table 1). Median cost per NICU hospitalization rose from $24,383 (IQR: $7670–$73,574) in 2017 to $29,234 (IQR: $9953–$85,152) in 2022. Median cost per NICU day also increased, from $3332 to $3779. Correspondingly, mean cost per NICU hospitalization rose from $73,633 (95% CI: 72,094, 75,171) in 2017 and $95,512 (95% CI: 93,570, 97,454). NICU costs represented between 18.2% and 28.1% of total hospital costs. Figure 2 summarizes the distribution of costs by gestational age over the 6-year study period. When stratified by gestational age, extremely preterm infants, though only 7.0% of admissions, accounted for 28.9% of total NICU costs, with a median cost per hospitalization of $275,208 (IQR: $75,232–$462,152) (Table 3). In contrast, term infants comprised 44.7% of admissions and 24.0% of costs, with a substantially lower median cost of $15,449 (IQR: $6,782–$41,746). NICU costs did not significantly change over time for term infants.

**Table 3:** NICU admissions, cost, length of stay by gestational age.

	Total cohort	Extremely preterm	Very preterm	Late preterm	Term	Unknown
**NICU Admissions**	234,571	17,042	36,608	47,627	104,938	28,356
**NICU Cost, per admission**, median (IQR)	$28,052 ($9,484, $83,028)	$275,208 ($75,232, $462,152)	$87,309 ($40,534, $165,654)	$30,833 ($12,937, $60,430)	$15,449 ($6,782, $41,746)	$15,391 ($4,781,$46,308)
**Length of Stay in days**, median (IQR)	10 (3,28)	84 (17,126)	34 (19,55)	12 (6,20)	5 (3,13)	7 (2,21)
**Length of Stay in days**, Mean (SE)	25.9 (0.1)	90.1 (0.6)	44.0 (0.2)	20.1 (0.2)	13.3 (0.1)	20.5 (0.2)
**% of NICU Admissions**	6.2^a^	7.2^b^	15.6^b^	20.3^b^	44.7^b^	12.1^b^
**% of NICU costs**	26.7^a^	28.9^b^	24.0^b^	14.9^b^	24.0^b^	8.1^b^

^a^NICU Admissions/Total Hospital Admissions.

^b^Gestational Age Group/NICU Admissions.

**Fig. 3:**
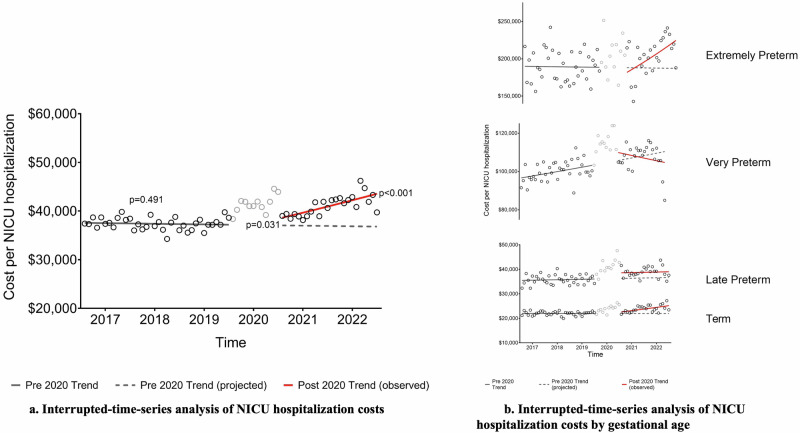
Interrupted-time-series analysis of NICU costs. Interrupted time-series analysis of NICU hospitalization costs overal (**a**) and stratified by gestational age (**b**).

ITS analysis revealed that NICU-related costs were relatively stable pre-pandemic (*p* = 0.491), there was a small but significant change in the model intercept ($38,646 [observed] vs $37,055 [projected], *p* = 0.031) after 2020 followed by an increase in NICU costs post-pandemic (*p* < 0.001) (Fig. 3a). Gestational age subgroup analysis suggested varied patterns across GA categories with the increased costs driven by the extremely preterm and term infants (Fig. 3b).

## Discussion

This analysis of NICU admissions across US children’s hospitals reveals an increase in overall admissions, higher acuity scores, and rising hospitalization costs between 2017 and 2022. The findings demonstrate increasing medical complexity, shifting demographic patterns, longer hospitalizations, and rising costs, trends that carry important implications for neonatal care delivery, planning, and policy. Rather than asserting broad policy implications, our results highlight the need for future population-based studies that evaluate NICU care delivery across hospital types, settings, and patient case mix.

### Demographic and clinical shifts

Over the 6-year study period, we observed notable shifts in the clinical and demographic profile of NICU admissions. The proportion of infants admitted with complex chronic conditions (CCC) increased, as did median illness severity scores. While full-term infants have traditionally been underrecognized in NICU cost studies, they consistently include nearly half of all NICU admissions in our cohort. Though race and ethnicity distributions shifted significantly, the decline in the proportion of infants with unknown race/ethnicity suggests this may be driven by improved data capture rather than true population change. Likewise, a reduction in unknown gestational age, from 18.1 to 10.9%, improved the precision of gestational age-based analyses. Among infants with known gestational age, the number of admissions increased by 10.3% for extremely preterm, 16.0% for very preterm, and 17.8% for late preterm infants, pointing to both enhanced reporting and increased population-level need. The significant increases in NICU admissions for late preterm infants, alongside the sustained need for complex medical care across all gestational age groups, further emphasize the necessity for adaptive healthcare strategies to meet the changing demands of neonatal care. The NICU population is becoming increasingly medically complex, and improvements in clinical documentation have enhanced our ability to track these trends.

### Outcome trends and hospital utilization

Longer hospitalizations and rising medical complexity were also evident. Across the study period, the proportion of short-stay NICU hospitalizations ( < 7 days) declined over time, while the volume of infants requiring longer stays ( > 31 days) increased. These findings suggest a growing cohort of infants requiring prolonged and intensive medical care, likely reflecting improved survival of high-risk infants, evolving clinical practices, and shifting thresholds for NICU admission [26]. Admissions from outside hospitals comprised 69% of the cohort, and most infants (89%) were discharged home, while 6% required transfer and 3.6% died in-hospital. The distribution of medically complex diagnoses also shifted: cardiovascular, gastrointestinal, and respiratory conditions became more prevalent across gestational age groups. Taken together, these outcome patterns indicate increased demand for high-resource neonatal care and reinforce the critical role of children’s hospital NICUs in managing complex, long-stay admissions.

### Cost implications and financial burdens

The financial implications of these trends are sizeable as NICU hospitalizations represent a significant portion of pediatric inpatient expenditures. While extremely preterm infants accounted for only 7% of admissions, they represented 29% of NICU-related costs, with median hospitalization costs reaching $275,208. Term infants, while less costly on average ($15,449), represented 45% of admissions and accounted for nearly one-quarter of NICU-related costs—highlighting a previously underappreciated source of spending. NICU costs per hospitalization and per day rose steadily across the study period, and the average cost per hospitalization in 2021 and 2022 exceeded pre-pandemic projections. Interrupted time series analysis confirmed a significant shift in cost trajectories following the COVID-19 pandemic, particularly for extremely preterm and term infants.

Comparatively, recent literature corroborates the high financial demands of neonatal intensive care, especially for preterm and medically complex infants. A 2021 study reported that NICU admissions accounted for 18% of infant hospitalizations, with an average spending of $71,158 per admission, and costs ranging from $4488 to $161,929, reflecting the variability in care complexity [27]. Another study focusing on preterm infants born at or before 30 weeks’ gestation found a median NICU admission cost of $77,132.90, with interquartile ranges between $48,338 and $126,680 [28]. Furthermore, children with medical complexity (CMC), though representing less than 1% of the pediatric population, are responsible for a substantial portion of healthcare expenditures, indicating that CMCs account for almost one-third of all child health spending, with rehospitalization comprising the largest proportion of subsequent costs (27.2%) [29].

These studies align with our findings, highlighting the significant financial resources required for the care of medically complex infants and confirms this population continues to drive pediatric hospital resource use. The continued increase in NICU admissions among infants with chronic conditions calls for investment in integrated care models (i.e., care coordination programs) that bridge inpatient and outpatient services to ensure smoother transitions post-discharge and address the social determinants shaping neonatal outcomes [30]. Rising NICU costs, particularly among high-acuity and term infants, highlight the critical need for hospital-level resource planning and greater attention to the economic impact of neonatal care.

Importantly, the interpretation of increased admissions and costs should be contextualized within the distinction between utilization and population-level need. While the economic concept of “demand” refers to actual use, this may not equate to the health system’s optimal or equitable provision of care. Moreover, although we observed rising direct hospital costs, these estimates are derived from charge data and do not reflect total family spending, reimbursement, or out-of-pocket expenses. As such, we cannot draw conclusions about family financial burden, and future studies using patient-reported outcomes and payer-level data are needed to assess these dimensions.

While NICUs have traditionally been associated with the care of preterm infants, our data indicate that full-term infants account for nearly half of admissions, underscoring the diverse and evolving nature of neonatal critical care needs at children’s hospitals. This finding is particularly relevant for term infants with complex conditions, for whom cost estimates have historically been limited. Additionally, this study provides a novel contribution by examining NICU costs as a proportion of total children’s hospital expenditures, offering a critical perspective on the financial impact of neonatal intensive care relative to overall hospital costs at US children’s hospitals. This lens is important given the increasing regionalization of neonatal care and closure of community pediatric beds, resulting in a significant shift in the care of term and late-preterm infants towards Children’s Hospital NICUs. These data provide important insights into high-acuity neonatal care that complements, rather than duplicates, national estimates by providing NICU-specific cost data for infants admitted to tertiary care hospitals. Understanding how admissions, complexity, and costs are evolving within children’s hospitals remains a critical step in assessing resource needs and ensuring equity in neonatal outcomes.

### Limitations

Our study has a few limitations. Research using administrative data has some degree of error and missingness of patient clinical data. For example, there were many encounters in the PHIS database where the infant had an unknown gestational age and while the change in unknown GA decreased by 7% over the study period, the unknown GA group may account for some of the trends seen in the gestational age categories during the study period. Secondly, our data is specific to children’s hospitals and likely underestimates NICU admissions overall, and the characteristics of neonatal admission in non-children’s hospitals may be different given the variation in level of care [12]. The objective of this study, however, was not to estimate national NICU costs but to characterize trends in a highly specialized subgroup that is underrepresented in other datasets. Lastly, given our study period covers the year(s) of the COVID-19 pandemic, its impact likely influenced some results. Even so, the data is still clinical meaningful as it provides trend information before and after the global pandemic. Further exploration is warranted to better identify the mechanisms driving higher costs during the pandemic that is not related to higher volumes of admissions.

## Conclusion

This study provides an updated analysis of NICU admissions and costs in U.S. children’s hospitals. Unlike prior research focused primarily on preterm infants, we show that term infants account for nearly one-quarter of NICU hospitalization costs, highlighting a critical gap in existing estimates. The substantial resources required to care for term infants with complex conditions underscore the need to include this population in future economic evaluations of neonatal care. Although this study did not explicitly examine the role of social determinants of health, our findings point to the importance of future work linking hospital- and population-level datasets to evaluate how factors such as insurance coverage, race/ethnicity, and neighborhood context shape variation in care, outcomes, equity, and costs. While NICU care is resource-intensive, it is important to note that most infants survive and experience positive developmental outcomes. Additionally, NICUs may contribute to overall hospital margin and sustainability, particularly within children’s hospitals; however, the financial implications of neonatal care must be interpreted within an ethical framework that prioritizes infant and family well-being, rather than revenue generation. Ultimately, these findings raise important questions for hospital leaders and researchers working to improve the efficiency, equity, and long-term sustainability of neonatal intensive care.

## Supplementary information

Supplemental Material

## Data Availability

The data that support the findings of this study may be available from Children’s Hospital Association, but restrictions apply to the availability of these data, which were used under license for the current study, and so are not publicly available.
