# Supplementary material for: Trends in admissions and costs for neonatal intensive care in US children’s hospitals, 2017–2022
Source: J Perinatol. 2026 Mar 17;46(6):1035–43. doi: 10.1038/s41372-026-02619-8 (PMC13290482; doi:10.1038/s41372-026-02619-8)
Supplement: Supplementary file 1 — Supplemental Material [file 41372_2026_2619_MOESM1_ESM.docx]

| **Supplemental Table. Example IDC-10 diagnosis codes for CCC Classification Domains**^17^ | |
| --- | --- |
| **Cardiovascular** | **Congenital or Genetic Defect** |
| Coarctation (Q25.1)  Tetralogy of Fallot (Q21.3)  Hypoplastic left heart (Q23.4) | Diaphragmatic hernia (Q79.0)  Trisomy 21, 18 (Q90.9, Q91.3)  Gastroschisis (Q79.3) |
| **Gastrointestinal** | **Hematologic or Immunologic** |
| Imperforate anus (Q42.3)  Hirschsprung's disease (Q43.1)  Atresia of esophagus with trachea-esophageal fistula (Q39.1) | Aplastic anemias (D60-D61)  Acquired immunodeficiency (B20-B24) |
| **Malignancy** | **Metabolic** |
| Neoplasms (C00-C9) | Tay-Sachs disease (E75.02)  Albinism, unspecified (E70.30) |
| **Premature and Neonatal** | **Neurologic** |
| Extreme immaturity (< 27 weeks) (P07.21 – P07.25)  Hypoxic-ischemic encephalopathy (P91.61 – P91.63)  Hydrops (P56.0) | Anencephaly (Q00.0)  Microcephaly (Q02)  Spina bifida (Q05.9)  Cerebral palsy, unspecified (G80.9) |
| **Renal & Urologic** | **Respiratory** |
| Congenital hydronephrosis (Q62.0)  Epispadias (Q64.0)  Potter's syndrome (Q60.6) | Congenital tracheomalacia (Q32.0)  Conjoined twins (Q89.4)  Choanal atresia (Q30.0)  Cystic Fibrosis: Meconium ileus in cystic fibrosis (E84.11) |
| Note: The CCC domains and diagnoses were not designed to be mutually exclusive and nor was it intended to prioritize one domain or diagnosis over another, as it is not a severity index. Often, the stratification by number of CCCs reflects the clinical complexity of infants with multi-system conditions and consistent with the tool's intended use. | |
